# Gene Expression upon Proliferation and Differentiation of Hematopoietic Cells with Ph Chromosome ex vivo

**Published:** 2012

**Authors:** N.I. Grineva, E.A. Duchovenskay, A.M. Timofeev, T.V. Akhlynina, L.P. Gerasimova, T.V. Borovkova, D.A. Schmarov, N.G. Sarycheva, N.M. Naydenova, A.R. Gavrichkova, L.Y. Kolosova, T.I. Kolosheynova, L.G. Kovaleva

**Affiliations:** Research Center for Hematology, Russian Ministry of Health and Social Development, Novy Zykovsky proezd, 4а, Moscow, Russia, 125167

**Keywords:** gene expression, regulation of cell proliferation and differentiation, cells containing Ph chromosome, chronic myeloid leukemia, RT-PCR, cell cycle, apoptosis

## Abstract

The genes*p53, mdm2, p21, c-myc,bcr/abl, bcr, bcl2, bax, *and* gapdh *participate in the regulation of cell proliferation and differentiation, apoptosis and cell distribution for the cell cycle* ex vivo *in the Ph^+^cells of chronic myeloid leukemia containing the Ph chromosome and*bcr/abl*oncogene. Expression of these genes correlates with regulation of cell proliferation and differentiation by alternating proliferation and maturation stages for three main Ph+cell types that occur under chronic myeloid leukemia. The*p53, p21, mdm2, *and* gapdh *genes overexpress in active proliferating myeloid cells in the cell cycle S+ G2/M phases and when the phases are coincident with the proliferation stage. Expression of these genes decreases to a considerable level under alternation of the Ph^+^cell proliferation and maturation stages and whenever the expression is greatly diminished under significant neutrophil accumulation and especially under repeated alternation of the stages. In the course of neutrophil maturation, gene expression levels decrease in the range of* gapdh > actin > c-myc, bcr/abl,p21 > p53 > bcl2 > bax.*The expression levels of these genes in neutrophils are lower than those in myelocytes and lower by an order of magnitude than that in the cells with a prolonged proliferation stage. The*Bcr/abl*expression gene under prolonged maturation and neutrophil accumulation is inhibited; however it is enhanced by 2–3 times for the proliferation stage with myelocyte accumulation. Minimal*bcr/abl*expression is observed under overexpression of*p53, mdm2, p21, c-myc,*as well as under cell maximum at the S and G2/M phases.* Bcr/abl*overexpression is observed under low expression of the*p53, p21, mdm2*genes. In the Ph^+ ^cells with a high P/D efficiency index (5–20), overexpression of the genes in the range of*bcr> gapdh>bcr/abl*, as well as a decreased expression of the*p53, bcl2, mdm2, p21<< gapdh *genes is observed for Ph^+^cells from the CML blast crisis and CML acceleration phase. Low control of cell proliferation and cell cycle by gene-regulators presumably promotes*bcr/abl*overexpression and activаtes the production of*bcr/abl+* cells. Apoptosis in the Ph^+ ^cells is induced by expression of the*bax > bcl2, р53, p21, c-myc andgapdh*genes. The blocking of Ph^+^cell apoptosis, neutrophil accumulation, and decrease in the expression of the* p53, mdm2 *and* p21, c-myc,bcr/abl *genes occur at the maturation stage.

## INTRODUCTION

Anomalies, translocations, inversions, deletions, and multiple mutations of chromosomes lead to the development of most leukemias ([1–5], and references therein). The Philadelphia chromosome (Ph) appears as a result of the chromosomal translocation t(9;22)(q34;q11) in a hematopoietic polypotent stem cell; this chromosome leads to the development of chronic myeloid leukemia (CML), as well as acute and chronic lympholeukemias. A chimeric oncogene *bcr/abl* encoding active tyrosine kinase р210/р185 that participates in CML pathogenesis is formed in cells containing the Ph chromosome (Ph ^+ ^ cells) due to reciprocal translocation of the 5’ fragment of the *bcr* gene and the 3’ fragment of the *abl* gene. Translocation results in the replacement of normal hematopoietic cells with Ph ^+ ^ cells. Numerous genes ( *bcl2, * a number of *stat* genes, and the genes regulating the cell cycle and apoptosis) participate in the cellular and molecular mechanisms of CML pathogenesis [1–57].

The ability of the *bcr/abl * oncogene to determine tumorgenic properties, enhance the viability, activate proliferation, and block apoptosis in Ph ^+^ cell lines has been thoroughly studied [9–7, 42–57]. The bcr/abl tyrosine kinase р210 was found to be capable of both suppressing apoptosis and making no contribution to it. The data relating to apoptosis blockage upon CML remain controversial [1–5, 42, 44, 45, 47 and our unpublished data]. The contribution of apoptosis to the proliferation and differentiation of Ph ^+^ cells had not been studied earlier. Our recent research demonstrates that apoptosis is dependent on the proliferation and maturation stages, as well on the type of Ph ^+^ cells derived from bone marrow (BM) and the peripheral blood (PB) of CML patients [Grineva *et al* .; unpublished data].

*Ex vivo* proliferation and differentiation of three main types of Ph ^+^ cells is regulated by alternating the cell proliferation stage (stage 1) and neutrophil maturation (stage 2). The proliferation rate is higher than the maturation rate at stage 1, whereas the maturation rate is higher at stage 2. The alternation of the stages and their rates maintains the optimal level of proliferation and differentiation efficiency in Ph ^+^ cells [1–4] and determines the wave-like regulation of these processes.

This study was aimed at putting the spotlight on the contribution of the expression of the genes that usually regulate proliferation and differentiation, apoptosis, and the cell cycle of normal hematopoietic cells to the regulation of these processes in Ph ^+^ cells. The kinetics of the expression of the *p53, c-myc, bcr/abl, mdm2, p21, bcl2, bax, * and * bcr * genes, as well as that of the control genes *gapdh * and * actin,* was studied. The ranges of gene expression kinetic curves and regularities of *ex vivo* proliferation, differentiation, apoptosis, and distribution in the phases of the cell cycle of Ph ^+^ cells isolated from CML patients were obtained.

CML Ph ^+ ^ cells consisting of 90% granulocytes are notable for their capacity to perform a complete proliferation and differentiation cycle, similar to that in the normal myeloid cells whose content is lower by an order of magnitude in the hematopoietic cell pool. This fact allows one to investigate the regularities of the regulation of proliferation and differentiation and their extrapolation onto normal haematopoietic cells.

## MATERIALS AND METHODS

Heparin (Flow, UK); Limphoprep, α-МЕМ medium (MP Biomedical, USA); DEPC, HEPES, Tris, fetal bovine serum (FBS), sodium citrate, lauryl sarkosyl (ICN, USA); trypan blue stain, *L* -glutamine and 2-mercaptoethanol (Serva, Germany); TRI reagent, guanidine thiocyanate (Sigma, USA); RQ1 RNase-free DNAse, RNasin, dNTP, bovine serum albumin (BSA), Taq polymerase, RT buffer, MuMLV reverse transcriptase (Promega, USA); penicillin and streptomycin (OAO Biochimik, Saransk, Russia); tabletted PBS (10 mM phosphate buffer + 0.13 M NaCl + 2.7 mMKCl, pH 7.4) (NPO EKO-servis, Russia) were used in this study.

Oligonucleotide primers ( *Table* ) were synthesized and purified by PAGE gel electrophoresis or HPLC by Sintol company (Moscow).

The Ph ^+^ mononuclear cells used for the study were prepared from the PB and BM of CML patients in the chronic phase, acceleration phase, and blast crisis phase before and under treatment. In CML patients, mononuclears are mostly represented by leukocytes and granulocytes; hence, we researched these cells. The characteristics of the Ph ^+^ cells and CML patients from whose PB and BM the mononuclears were isolated are given in [2–5]. The types of *bcr/abl* mRNA (b3a2, b2a2 or e1a2) in the Ph ^+^ cells were determined by RT-PCR [2, 5].

The methods for isolating mononuclear cells and analyzing the proliferation and differentiation of Ph ^+ ^ cells were previously described [1–6]. Suspension (0.8–1.2) × 10 ^6^ cells/ml was incubated with an α-МЕМ medium containing 10–20% FBS, 2 mM *L* -glutamine, 10 ^-4^ M 2-mercaptoethanol, 100 U/ml penicillin, and 50 U/ml streptomycin, and 25 mM HEPES-NaOH pH 7.2–7.4 were cultured under strictly identical conditions; samples were collected for further analysis.

The degree of apoptosis and distribution of cultured Ph ^+^ cells over the phases of the cell cycle were analyzed cytofluorometrically [1–4] in the granulocyte gate on an EPICS-XL flow fluorimeter. Ph ^+^ cell samples (5,000 cells each) isolated from BM and PB in a Ficoll density gradient and the samples collected during the cultivation were centrifuged for 7 min at 600 g and 4 ^о^ С, washed with PBS, and fixed dropwise adding cooled 70% ethanol during 30 min at 4 ^о^ С. Prior to measurements, the cell suspension was washed with PBS and centrifuged; the precipitate was incubated in 0.5 ml PBS supplemented with propidium iodide (5 µg/ml) and RNase A (50 µg/ml) for 30 min at room temperature in the dark. The measurements were carried out in an EPICS-XL flow fluorimeter. The cells in the granulocyte gate were analyzed using forward-scattered light (FSC) and side-scattered light (SSC) with simultaneous registration of the FL2 fluorescence based on pulse amplitude and area (this allowed eliminating aggregated cells, conglomerates, and debris) in the linear and logarithmic scales. Apoptotic cells were detected simultaneously. FL2-H particles with hypodiploid DNA located as a separate peak leftward of the peak of diploid cells (a decrease in cell size not higher than 2 orders of magnitude) were considered to be apoptotic. The percentage of apoptotic granulocytes was estimated within the granulocytic gate containing no cell debris. The DNA histograms from the same cell samples were analyzed for cell cycle phase distribution (S, G2/M) using specialized software (SFIT method) [7, 10]. Samples containing 10 ^6^ cells were used to isolate cellular RNA. Each sample underwent lysis by guanidine isocyanate according to [11], with small modifications [5].

**Table 1:** Table. Oligonucleotide primers for RT-PCR

mRNA,target	Primers Sequence 5’ → 3’, Gene localization GenBank Acc.no		PCR fragment,bp
	Outer primers, 56оС annealing,1st round	Inner primers, 60оС annealing, 2nd round	
*bcr/ablb3a2,b2a2*	TGGATGAACTGGAGGCAG NM_005157 (342–361 bp, 20b) TCA CAG GCG TGA TGT AGT T NM_007313 (835–854 bp, 20b) NM_004327 (2896–2913 bp, 22b) (90% гомология)	GGAGCTGCAGATGCTGACCAAC NM_004327 (3227–3248 bp, 22b) GCTTCACACCATTCCCCATTNM_007313 (3477–3496 bp, 20b) NM_005157 (289–308 bp, 20b)	378 b3a2,303 b2a2
* bcr*	TGGATGAACTGGAGGCAG NM_004327 (2896–2913 bp, 22b)CAGTTTGGCTCAGCTGTGTCCCNM_004327 (3448–3469 bp, 22b)	GGAGCTGCAGATGCTGACCAAC.NM_004327 (3227–3248 bp, 22b) CAGTGGCTGAGTGGACGATGANM_004327 (3340–3360 bp, 21b)	134
* mdm2*	ATGTGCAATACCAACATGTCNM_002392 (297–317 bp, 20b)TAGGGGAAATAAGTTAGCAC NM_002392 (1470–1492 bp, 20b)	CAAGAACTCTCAGATGAAGATG NM_002392 (1092–1114 bp, 22b) TTGATGGCTGAGAATAGTCTTC NM_002392 (1470–1492 bp, 22b)	401
*p53*	ATTGGCAGCCAGACTGCCTT NM_000546 (219–238 bp, 20b)GGAACAAGAAGTGGAGAATGNM_000546 (1434–1453 bp, 20b)	AGCTACTCCCCTGCCCTCAA NM_000546 (624–643 bp, 20b) GTCTTCCAGTGTGATGATGG NM_000546 (1009–1028 bp, 20b)	405
* gapdh*	GCTTGTCATCAATGGAAATCNM_002046 (300–319bp, 20b)CACGATACCAAAGTTGTCATGNM_002046 (595–615 bp, 21b)		316
*bcl2*		TGTGGAACTGTACGGCCCCAGCATGC NM_000633 (1087–1113 bp, 27b) GCCTGCAGCTTTGTTTCATGGTACATC NM_000633 (1286–1312 bp, 27b)	226
* bax*		CATCAGGGACTCAGTTGTNC_000019 (522–540 bp, 19b)CACTCCTCAAATCTGTGCCANC_000019 (764–783 bp, 20b)	262
* p21*		GCCGGAGCTGGGCGCGGATT NM_07846(42–61 bp, 20b) GGCTTCCTCTTGGAGAAGAT NM_07846 (707–726 bp, 20b)	685
*actin,beta*(ACTB)		GCGGGAAATCGTGCGTGACATTM10277complete CDS (2280–2301 bp, 22b)GATGGAGGTTGAAGGTAGTTTCGTG M10277 complete CDS (2583–2606 bp, 24b)	327
* c-myc*	GAGGCTATTCTGCCCATTTG NM_002467 (440–459 bp, 20b)GGCAGCAGCTCGAATTTCTTNM_002467 (721–740 bp, 20b)		301

The samples were treated with DNase according to [5]. RNA isolated from the sample (10 ^6^ cells) was annealed with 50 ng of a hexamer mixture in 8 µl of water (70 ^о^ С, 10 min). cDNA was synthesized during 1 h at 37 ^о^ С in 25 µl of a RT buffer (Promega) containing 2.5 µM of each dNTP, 20 AU RNasin (Promega), and 20 AU MuMLV reverse transcriptase (Promega). The cDNA solution was stored at –70 ^°^ С and immediately used to carry out PCR.

Transcription of the *p53, c-myc, bcr/abl, mdm2, p21, bcl2, bax, * and *bcr * genes and the control genes *gapdh * and *actin * was analyzed by RT PCR. RT PCR was carried out using specific primers on RNA isolated from each probe ( *Table* ) using one or two rounds.

PCR was carried out in 25 µl of a solution containing the PCR buffer (50 mM Tris HCl pH 8.9, 16 mM (NH _4_ ) _2_ SO _4_ , 10 mM 2-mercaptoethanol, 50 µl EDTA, 0.14 µg/ml BSA), 2–5 µl of a cDNA solution, 200 µM of each dNTP, 2.5 AU Taq polymerase (Promega), and 75 ng of each primer ( *Table* ). PCR (30 cycles) was carried out in accordance with the following scheme: denaturation – 1 min, 94 ^o^ С; annealing – 1 min, 56 ^о^ С for the 1st round and 60 ^o^ С for the 2nd round; and synthesis – 3 min, 72 ^o^ C. cDNA probes from the * bcr, p53, mdm2 * and *bcr/abl* genes were annealed at 56 and 60 ^o^ C for the outer and inner primers, respectively ( *Table* ). PCR products were analyzed by electrophoresis in 6% PAGE. Gels were stained with ethidium bromide (1 µg/ml). The current fluorescence intensity of the amplified fragments ( *Jt* ) was determined via computer densitometry using the Scion Image software with allowance for the volume of the RT PCR and electrophoresis probes.

Gene expression was judged based on the results of the RT-PCR carried out using the total RNA of Ph ^+^ cells with the primers specified in *Table* . The mRNA expression level was assessed based on the fluorescence intensity ( *Jt* ) of the bands corresponding to the cDNA amplification products. The level of expression of *gapdh* and/or *actin * mRNA in the same probe was used as an internal reference.

Expression of *bax * mRNA isoforms [9] was analyzed using primers for the amplification products of the *bax* RNA alternative splicing of intron I ( *Table* ); the accumulation of its PCR fragment correlates with the expected expression of the *bax* , *bcl2 * and other genes, as well as with apoptosis kinetics ( *Figs. 1–9* ).

The kinetic plots of the gene expression, proliferation, differentiation, apoptosis, and distribution of the Ph ^+ ^ cells over the phases of the cell cycle were presented in a polynomial approximation. The alteration of the fluorescence intensity ( *Jt* ) was used to determine the positions of the peaks of RNA expression and their maximum; *Jt/Jgapdh * was used to assess the relative levels of mRNA expression. Hence, these results can be compared to the data that were obtained by measuring of the expression levels in separate probe, e.g. by a method widely used in other studies.

A polynomial approximation to the 6th power was used to process the curves of gene expression, cell proliferation and differentiation on the grounds that the curves are of a wave-like character with several maxima and minima and obey neither the logarithmic nor exponential law. The following advantages and limitations of the polynomial approximation were taken into account. The optimal number of generalized data is equal to the approximation power minus one. Approximation was considered reliable based on the accuracy of the experimental data ± 10% given in [1–6] (R ^2 ^ ≥ 0.81–1). The number of approximated points could be higher than the approximation power index by one or two. The points belonging to the first growth period (five to eight points in our experiments for a time interval of 8–10 days) are of special importance for characterization of the kinetic curves. Probing after 24 h upon *ex vivo* CPD corresponds to the expected time of development of the cell cycle in animal cells *in vivo* , which is close to 24 h. One or two points were missing at the peak vertex if the kinetics was known (calculated and predicted by software based on the peak start) to allow one to plot the whole kinetic curve.

A morphological analysis was used to plot the kinetic curves of the proliferation and differentiation of Ph ^+^ granulocytes and their subpopulations, the myeloid cells (blasts, promyelocytes, myelocytes, metamyelocytes, segment and band neutrophils). Cell composition was analyzed using smears (three areas for each smear, each area containing 100 cells). The concentration of cell subpopulations in the probes was determined based on their content in the smears recalculated for 10 ^6^ cells/ml of the original suspension [1–6].

The kinetic curves of the P/D efficiency index (the ratio between the neutrophil proliferation and maturation rates) were obtained as ratios between the accumulation of immature proliferating cells, P (blasts, promyelocytes, myelocytes), and the accumulation of neutrophils maturing without dividing, mature cells, D (metamyelocytes, bands and segments) according to [1–4].

## RESULTS

The kinetic curves of the gene expression levels (GEL) of *p53, p21, c-myc, bcr/abl, mdm2, bcl2, bax, bcr* , which participate in the regulation of the cell cycle [14, 24, 28, 45–48, 52, 58, 59], apoptosis [3, 14, 16–22, 28, 42, 47, 49, 50, 56, 58, 60], proliferation and differentiation, were obtained by cultivation of Ph ^+ ^ mononuclear cells consisting of 95% myeloid Ph ^+ ^ cells; i.e., upon CML-affected myelopoiesis [1–3, 24, 26–28, 42, 43, 46, 48, 51–54, 57–68].

The kinetic curves of the expression of the *c-myc, p53, bcr/abl, mdm2, p21, bcl2, bax, gapdh, actin, bcr* genes were compared to those of the regulation of the proliferation and differentiation of three main types of myeloid Ph ^+^ cells and their apoptosis and distribution in the phases of the cell cycle. The GEL and CPD curves were obtained using the same probe for each assay.

mRNA expression levels were assessed based on the fluorescence intensity ( *Jt* ) of the corresponding RT-PCR products of the genes under study. The *gapdh * and * actin* genes were used as the control. The * Jt * value was used to estimate changes in gene expression and peak positions. The values of *Jt/Jgapdh* allow one to estimate the ratio between gene expressions; however, the positions of the peaks, as well as their maxima and minima, are noticeably altered due to the changes in the *gapdh* expression. Early changes in the expression level of *gapdh * were also observed in other studies [55, 56].

**Fig. 1:**
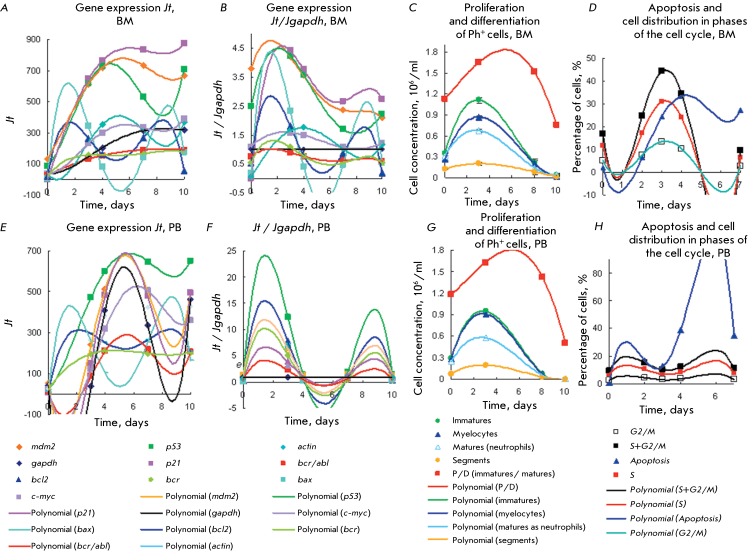
Expression of *p53, mdm2, p21, c-myc,* *bcr/abl, bcr, bcl2, bax, gapdh , actin * genes(a *, b, e, f* ) for CML Ph ^+^ cells of type 1 represented by a prolonged proliferation stage with moderate proliferation efficiency. Comparison of kinetic plots for the expression levels of these genes with the kinetic plots of proliferation and differentiation ( *c,g* ), apoptosis and cell distribution in the cell cycle ( *d, h* ). Kinetic plots are assayed in the same probes for every process of Ph ^+ ^ cells from BM ( *a–d* ) and PB ( *e–h* ). Gene expression levels (GEL) are given as fluorescence units *Jt* ( *a, e* ) of total RNA from10 ^6^ cells estimated by RT-PCR and as the *Jt /Jgapdh * ratio *(b, f).* PD of Ph ^+ ^ cells ( *c, g* ), apoptosis and cell distribution in cell cycle ( *d, h* ). There are [immature] > [mature] cells and P/D index 1.2–1.8 on days 0–10. Polynomial approximation to the 6th power.

**Fig. 2:**
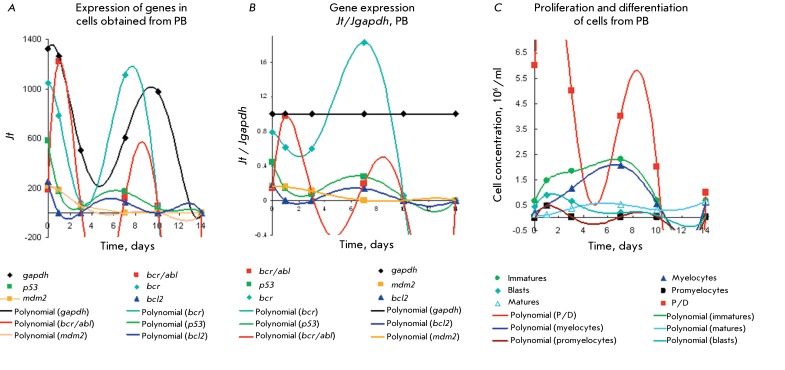
Gene expression levels of * p53, mdm2, bcr/abl, bcr, bcl2, gapdh* ( *а, b* ) for type 1 Ph ^+^ cells from PB with prolonged proliferation and a high efficiency P/D index =5–12 in comparison with the kinetic plots for proliferation and differentiation ( *c* ). Details are identical to those in Fig. 1. *Jt* ( *а)* and *Jt / Jgapdh (b).* Duration of the proliferation stage with [immature] > [mature] cells is 14 days.

**Fig. 3:**
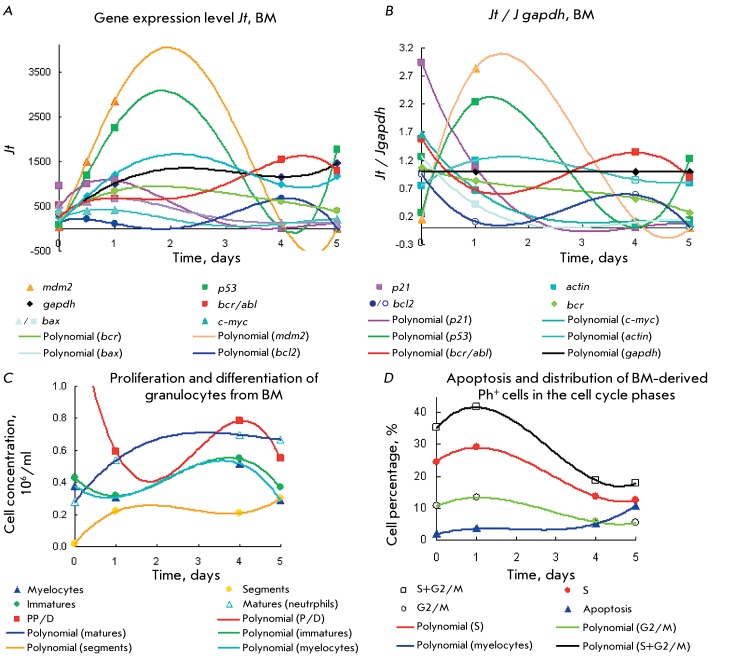
Expression of the *p53, mdm2 * and * p21, c-myc,* *bcr/abl, bcr, bcl2, bax, gapdh, * and * actin* genes ( *a, b* ) for CML Ph ^+ ^ cells of type 2 from BM with prolonged maturation stage and a low P/D efficiency index ≤ 1 and [matures] >[immatures]. Comparison of the kinetic plots for the gene expression level ( *a,b* ) with those for proliferation and differentiation ( *c* ), apoptosis, as well as with cell distribution in cell cycle ( *d* ). Details are identical to those in Fig. 1. *Jt* ( *a* ) and *Jt / Jgapdh (b)* .

**Fig. 4:**
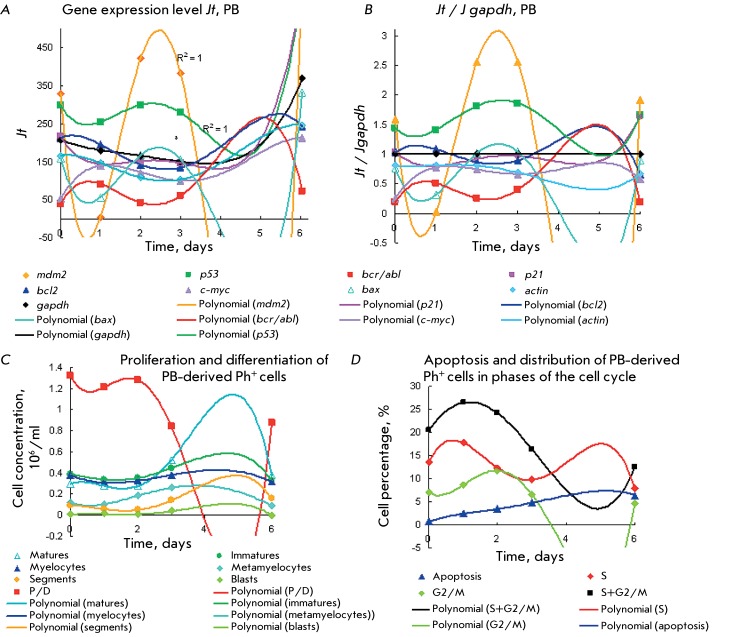
Kinetic plots for the gene expression levels of the *p53, mdm2, p21, c-myc,* *bcr/abl, bcr, bcl2, bax, gapdh, * and * actin* genes( *a , b* ) for CML Ph ^+ ^ cells of type 3 from PB with stage alternating according to scheme 1/2. Comparison with the plots for proliferation and differentiation ( *c* ), apoptosis and cell distribution in the cell cycle ( *d* ). Details are identical to those in Fig. 1. *Jt* ( *a)* and *Jt / Jgapdh (b).* Proliferation stage with [immatures] > [matures] on days 0–3. Maturation stage with [matures] > [immatures] cells occurred on days 3–6.

**Fig. 5:**
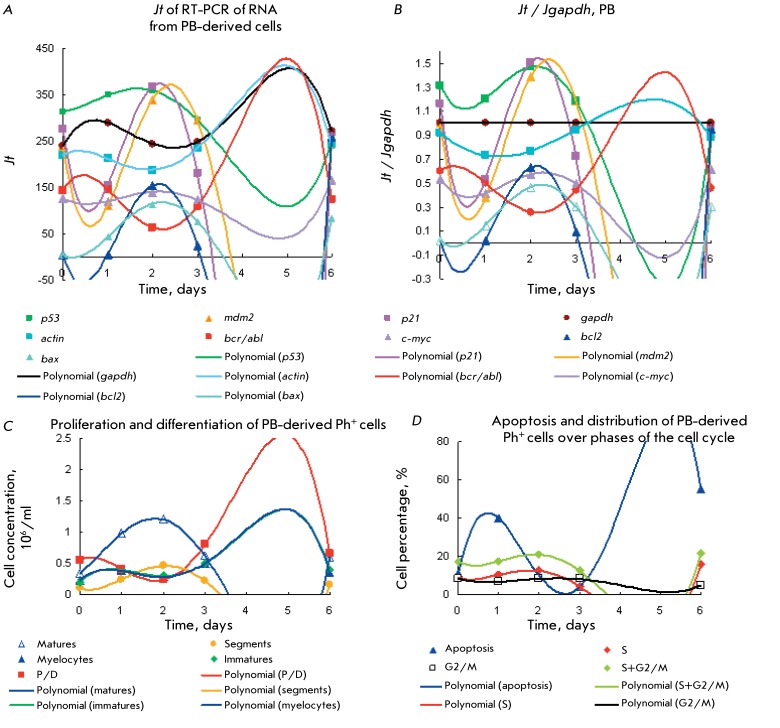
Gene expression levels of *p53, p21, mdm2, c-myc,* *bcr/abl, bcr, bcl2, bax, gapdh, actin * ( *a , b* ) for CML type 3 Ph ^+ ^ cells from BM with stage alternating according to scheme 2/1/2. Comparison with the kinetic plots for proliferation and differentiation ( *c* ), apoptosis and cell distribution in the cell cycle ( *d* ). Details are identical to those in Fig. 1. *Jt* ( *a)* and *Jt / Jgapdh (b).* Maturation stage with [matures] > [immatures] occurred on days 0–3 and 6. Proliferation stage with [immature] > [mature] cells occurred on days 3–6.

**Fig. 6:**
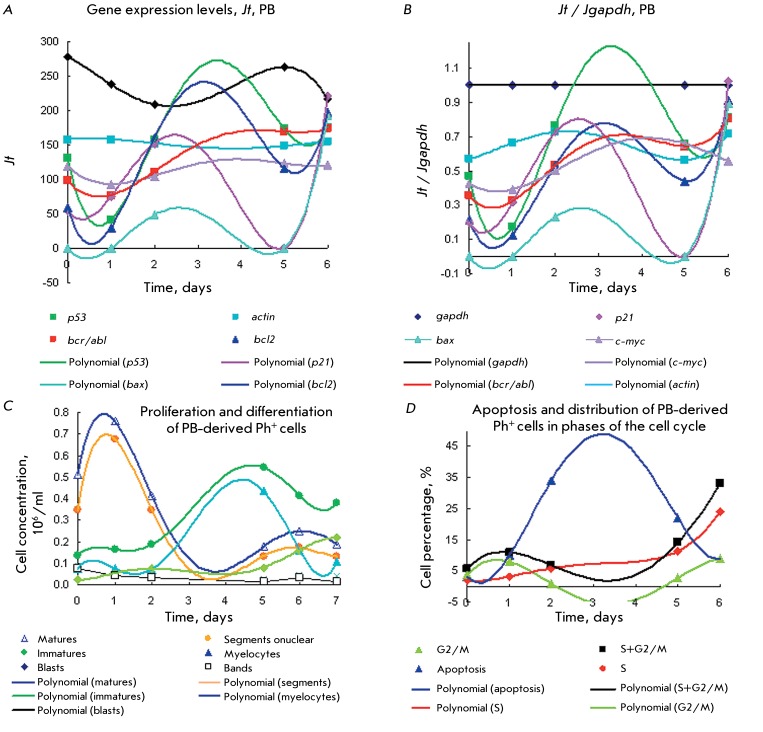
Gene expression levels of *p53, p21, c-myc,* *bcr/abl, bcr, bcl2, bax, gapdh, actin * ( *a , b* ) for CML type 3 Ph ^+ ^ cells from BM with stage alternating according to scheme 2/1/2 in comparison with the kinetic plots for cell proliferation and differentiation ( *c* ), apoptosis and cell distribution in the cell cycle ( *d* ). Details are identical to those in Fig. 1. *Jt* ( *a)* and *Jt / Jgapdh (b).* Maturation stage with [mature] > [immature] on days 0–3. Proliferation stage with [immature] > [mature] cells occurred on days 3–6.

**Fig. 7:**
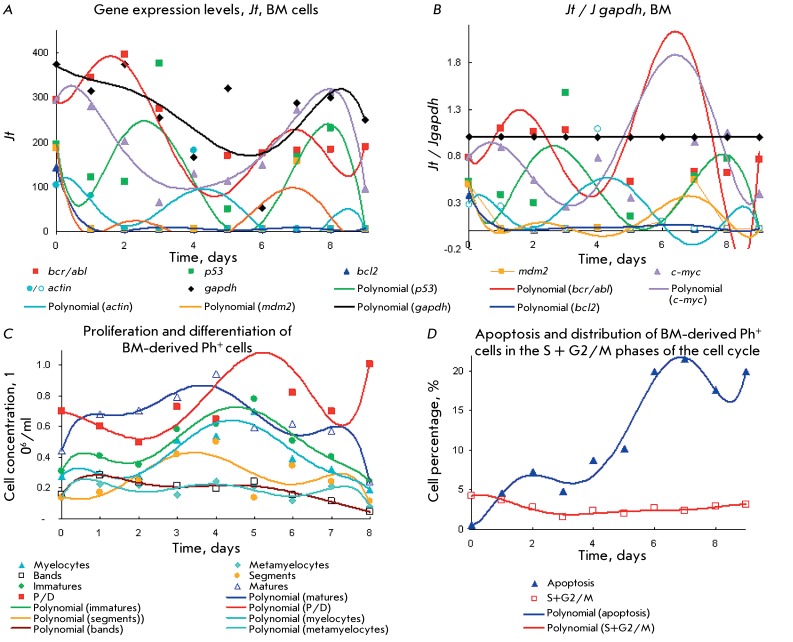
Expression levels of the *p53, mdm2, c-myc,* *bcr/abl, bcr, bcl2, gapdh, * and * actin* genes( *a, b* ) for CML type 3 Ph ^+ ^ cells from BM with stage alternating according to scheme 2/1/2. Comparison with the kinetic plots for cell proliferation and differentiation ( *c* ), apoptosis and cell distribution in the cell cycle ( *d* ). Details are identical to those in Fig. 1 *. Jt* ( *a)* and *Jt / Jgapdh (b).* Maturation stage with [matures] > [immatures] cells on days 0–5 and days 6–8. Proliferation stage with [immatures] > [matures] cells occurred on days 5–6 and day 8.

**Fig. 8:**
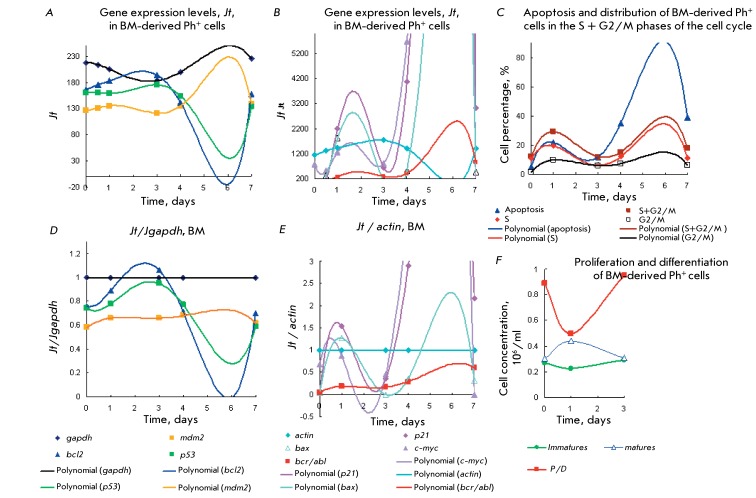
Kinetic plots for the gene expression levels of *p21, c-myc,* *bcl2, p53, mdm2, bcr/abl, bax, gapdh, actin* ( *a, b, d, e* ) for type 3 CML Ph ^+ ^ cells from BM with stage alternating according to scheme 2/1 in comparison with the kinetic plots for apoptosis and cell distribution in the cell cycle ( *c* ), as well as that for cell proliferation and differentiation ( *f* ). Details are identical to those in Fig. 1. *Jt* ( *a, b)* and *Jt / Jgapdh * ( *d* ), *Jt / Jactin* ( *e* ). Maturation stage with [mature] > [immature] cells occurred on days 0–3. Proliferation stage with [immature] > [mature] cells occurred on days 3–7.

**Fig. 9:**
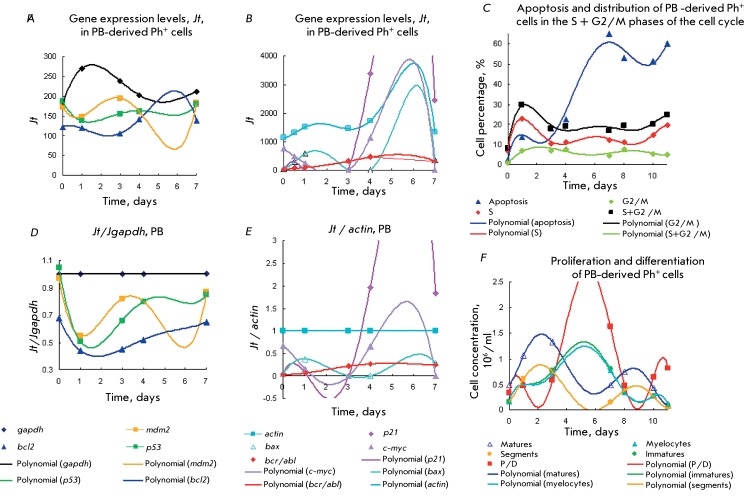
Gene expression of *p21, c-myc,* *bcl2, p53, mdm2,* *bcr/abl, bcl2, bax, gapdh, actin* ( *a, b, d, e* ) in comparison with the kinetic plots for apoptosis and cell distribution in the cell cycle ( *c* ), as well as that for cell proliferation and differentiation ( *f* ) for CML type 3 Ph ^+ ^ cells from PB with stage alternating according to scheme 2/1/2/1. Details are identical to those in Fig. 1. *Jt* ( *a, b)* and *Jt / Jgapdh * ( *d* ), *Jt / Jactin* ( *e* ). Maturation stage with [matures] > [immatures] cells occurred on days 0–4. Proliferation stage with [immatures] > [matures] cells occurred on days 4–7.

The kinetic curves of gene expression were compared to the regularities of the proliferation and differentiation of the granulocyte populations, the apoptosis and distribution of Ph ^+^ cells in the phases of the cell cycle, with alternating the proliferation and maturation stages, which regulate the P/D index efficiency of these processes [1–4] and are given in a polynomial approximation. The regularities of the proliferation and differentiation of Ph ^+^ cells have already been studied [1–6]; the polynomial approximation of these curves is considered here, since they fail to obey either the logarithmic or exponential law, and the kinetic curves corresponding to these dependences have several maxima and minima ( *Figs. 1–9* ).

According to [1–4], the regularities of the cell distribution over phases of the cell cycle, apoptosis level, and P/D efficiency index for three types of Ph ^+^ cells obtained from CML patients vary. The cell types, their proliferation, and differentiation differ by the sequence of stage alternations, as well as the number and duration of the stages. This study provides evidence to support the assumption that gene expression shows features of the regulation of the proliferation and differentiation of three types of Ph ^+^ cells, as well as their apoptosis and distribution over the phases of the cell cycle.

**Gene expression upon proliferation and differentiation of type 1 Ph ^+^ cells **

Type 1 Ph ^+^ cells are characterized by a prolonged proliferation stage (stage 1) at a rate higher than the maturation rate; the concentration of immature cells is higher than that of mature cells for an appreciably long time; P/D ^1^ index ≥ 1–20. These cells are notable for their enhanced accumulation of myelocytes, promyelocytes, and blasts with a small accumulation of neutrophils maturating without dividing, and active apoptosis of neutrophils [1–3].

Figures  *1A–H * show the kinetic plots of the gene expression, proliferation and differentiation of type 1 Ph ^+^ cells obtained from the BM and PB of CML patients with a moderate proliferative potential and a P/D index = 1–5. It is clear that the peaks with maximum and minimum gene expression in BM cells are clustered in three zones. Based on the peak area, gene expression in these zones can be divided into active and moderately active. Active expression of *bcl2* and *bax * genes can be seen in zone 1 (on days 1–2). The second zone within the range of days 2–7 is characterized by a wide peak of overexpression of the *p53, mdm2* ,and *p21* genes with the maximum on days 3–5 ( *Figs. 1A–D* , BM cells). 

The maximum expression of the *p21, mdm2, p53, actin, gapdh, and c-myc* genes decreases to a different extent within the same range to attain its minimum on days 8–9. The *c-myc, bcr/abl, gapdh, actin, and bcr * genes are expressed less actively in the second zone. All the genes, except for *bcr/abl,* have two expression minima: on days 1–2 and 8–9. In Ph ^+ ^ cells obtained from PB, the *р53* , *р21, mdm2, c-myc, bax,* and *bcl2 * genes are overexpressed in a similar manner; however, the peaks of expression of *р21, mdm2, c-myc * and *gapdh* are noticeably narrower ( *Figs. 1E,F* ).

Overexpression of *р21, mdm2 * and *р53* attains a maximum under cell proliferation and differentiation in accordance with cell distribution in the S and G2/M phases; i.e., it occurs in actively proliferating myeloid precursor cells. Expression of these genes decreases to some level to the end of the proliferation and differentiation cycle, with cell death on days 6–7 ( *Fig. 1C,G* ); it increases again on days 7–8. Meanwhile, expression of *c-myc, bcr/abl * and * gapdh * ismoderate. The concentration of proliferating (immature) cells is considerably higher than that of neutrophils (mature cells). Throughout the processes of proliferation and differentiation, the accumulation rate of proliferating cells is higher than that of maturating neutrophils; all cells have a common gene expression maximum corresponding to a high content of immature cells and a rather low content of neutrophils.

Active expression of *p53, mdm2, p21 * ( *c-myc, * to a lower extent) correlates with changes in cell concentration, cycle regulation, and cell apoptosis on days 3–4 and 7–10 ( *Figs. 1A–C, E–G* ). Overexpression of *p21, p53, mdm2 * and moderate and low expression of the other genes ( *actin, c-myc, gapdh, bcr,* and * bcr/abl* ) in Ph ^+^ cellsderived from BM should be regarded as gene expression in proliferative pool cells, which actively accumulate in the G1 and S phases of the cell cycle on days 3–4. The G1 phase including the synthesis of cyclins and kinases, formation of their ensembles, and phosphorylation of the Rb protein with the participation of p21 and the proteins responsible for passing the control points of the G1/S transition presumably occurs during this period [23, 24, 58, 64, 67–71]. This stage is accompanied by *р53* overexpression, which means that p53 fully performs its functions; i.e., it regulates transcription, cell cycle and its control points, differentiation, and apoptosis [10–16].

Maximum apoptosis of the cells prepared from bone marrow (~30%) is observed on day 4 and further slightly decreases. Minimum apoptosis is revealed 24 h after a rapid decrease in the beginning. Apoptosis intensity is higher in cells obtained from peripheral blood; it has two GEL maxima on days 1 and 5–6 and a minimum on days 2–3 ( *Figs. 1,3* ). This does not correlate with GEL of *bcl2 * and *bax* , which are responsible for apoptosis [13, 14, 16–22].

Expression of *bcl2* and *bax * in BM cells is characterized by two peaks with maxima on day 2 and a minimum on days 4–5, which is inconsistent with the apoptosis maxima in cells derived from BM and PB ( *Figs. 1D,H* ). It is a known fact that apoptosis can also be stimulated by actively expressed genes *p21, p53, gapdh, c-myc* [10–28, 32, 34, 49–51, 55, 56, 67–69]. The p21 protein inhibits cyclin-dependent kinases and mediates a number of p53 functions. Expression of *p21* is responsible for cell growth delay during the G1 phase, regulation of the cell cycle, and apoptosis [23–28, 64, 67, 68, 71]. If *p21* overexpression does not cause cell growth delay during the G1 phase, additional p21 molecules induce apoptosis and/or differentiation termination [24, 64, 68]. Apoptosis activation in response to *p21* expression occurs during this phase on day 4, provided that *bcl2 * and * bax * are not expressed ( *Figs. 1A–D* ).

The apoptosis level in PB-derived Ph ^+ ^ cells at the second peak with a maximum on days 5–6 is significantly higher than that in BM-derived cells ( *Figs. 1D,H* ). Comparison of the GEL ( *Fig. 1* ) reveals a similarity in the expression of the *р53, bcl2, * and * bax* genes in BM and PB cells and a narrower expression peak in PB cells. However, activation of gene expression *bax* **>** *bcl2 * with maxima on days 5–6 is absent in BM cells and does not match the second apoptosis peak observed on day 4. It is assumed that *р21* (which regulates apoptosis, according to [28, 57, 60]) participates in the regulation of this peak in BM cells. *gapdh* is simultaneously overexpressed in PB cells (the expression maximum is observed on days 4–6). Expression of *gapdh* and apoptosisin BM-derived Ph ^+ ^ cells increase several-fold ( *Figs. 1D,H* ).

Overexpression of *mdm2 * is associated with the functions of this transcription factor, which modulates the properties of a number of genes and interacts with various growth factors and transcription factors. The mdm2 and p53 proteins mutually interact and negatively regulate the expression of each other [29–36]. Overexpression of *mdm2 * presumably modulates the functions of p53 and p21, regulates the duration of the S and G2/M phases of the cell cycle, and enhances the proliferative potential of Ph ^+ ^ cells at a weak level of *bcr/abl* expression.

The tumor suppressor p53, which is activated by genotoxic and cellular stress, protects instable cells via the expression of the genes that trigger the cell cycle and inhibit proliferation, blocking apoptosis, and repairing DNA. Meanwhile, p53 and mdm2 activate each other and are simultaneously either stabilized or degraded. Stress-induced activation via a feedback mechanism results in the activation of *р53 * and *mdm2* [31–36] and protects cells against death. The interaction between *р53* and *mdm2* is attested by the coincidence of their kinetic plots ( *Figs. 1A,D,E,H* ) with the maximum of cell accumulation during the S and G2/M phases. *Mdm2* overexpression can be attributed to the activation of the delayed cell transition to the S and G2/M phases of the cell cycle on day 6 ( *Fig. 1H* ). Mdm2 is known to stimulate uncontrolled cell transition to the S phase [29]. Furthermore, overexpression of mdm2, which directly interacts with the *p53* and *p21 * promoters, results in uncontrolled cell transition to the S phase and their transformation [24, 29–31, 67, 68, 71].

The *bcr/abl* expression is known to activate the proliferation of Ph ^+^ cells [43–48]. In this study, Ph ^+^ cells derived from BM and PB were characterized by a very low level of *bcr/abl * expression, which was significantly lower than that of *р53,* *mdm2, p21* ,and * c-myc* . Rather low levels of *bcr/abl * expression lie within the zone of the maximum expression of *р53,* *mdm2, p21* , and even * c-myc * on days 3–10, which is in agreement with proliferation and differentiation efficiency values that are low for type 1 Ph ^+ ^ cells (P/D indices = 1.2–1.8–0.8). Expression of *bcr/abl* in PB cells is higher to some extent compared to that in BM cells. A maximum (day 5) and two minima (days 1 and 9) were observed in PB cells. In BM cells, *bcr/abl * expression slowly increases by days 4–10. These differences do not affect the P/D indices, attesting to the fact that the proliferation and maturation rates in cells derived from BM and PB are comparable. Low *bcr/abl* expression with high cell content during the S and G2/M phases can presumably be attributed to the suppression of *bcr/abl * upon overexpression of *p53, p21, mdm2,* *c-myc* , the main regulators of the cell cycle [10–16, 23–28, 31–36, 51–54, 67, 68]. Expression of these genes is also required for the proliferation of myeloid cells and termination of their differentiation. A decrease in the expression level can be in agreement with the decreasing concentration of immature dividing cells.

It is clear that the peak representing gene expression in PB-derived Ph ^+^ cells is narrower than that for BM-derived Ph ^+^ cells ( *Figs. 1D–H* ). Expression of *р53, bcl2,* and * bax * in PB and BM cells begins immediately and occurs in a similar manner, attaining its maximum on days 2 and 9 ( *bcl2* , *bax* ) and on day 5 ( *p53* ). In PB-derived cells, the *p21, mdm2,* and * c-myc* genes are expressed with a 3-day delay; the maximum level of expression corresponds to days 5–6. A rapid decrease in expression with a higher apoptosis peak is subsequently observed in these cells compared to that in BM cells ( *Figs. 1D,H* ). It is clear that maximum expression of each individual gene ( *p21, р53, mdm2 * and *c-myc* ) corresponds to the maximum content of cells derived from BM and PB during the S and G2/M phases ( *Figs. 1D,H* ). PB-derived cells are presumably synchronized to a larger extent compared to BM-derived cells.

Based on the *Jt/Jgapdh* ratio ( *Fig. 1B* ), one can assume that expression of the genes associated with the proliferation of BM-derived cells decreases for the range *mdm2 ~ p21 ~ p53 > actin ~ c-myc > gapdh ~ bcr/abl ~ bcr* . A 4.5-fold decrease in GEL corresponding to the peak maxima in BM-derived cells compared to that for *gapdh * is observed. In PB-derived cells, *gapdh * overexpression is combined with an abrupt decrease in the expression levels of other genes; thus, it makes no sense to use the *Jt* / *Jgapdh* coordinates for comparison.

It is clear from *Fig. 1* that the expression of a number of genes (including *bcr/abl* ) correlates with the regularities of the proliferation and differentiation, apoptosis, and distribution of Ph ^+ ^ cells in the cell cycle phases. The correlation between the maximum accumulation of proliferating and differentiating cells and gene expression means that the *p21, mdm2, p53, c-myc, bcr, bcl2, * and *bax* genes participate in the regulation of proliferation, differentiation, and apoptosis of type 1 Ph ^+ ^ cells. However, expression of these genes cannot be linked to various subpopulations derived from type 1 Ph ^+^ cells, since they are produced by a single peak with the same time maximum.

Overexpression of genes *bcr > gapdh > bcr/abl * with two maxima on day 1 and days 7–10 and a minimum on days 4–5 can be observed for a sample of type 1 Ph ^+^ cells derived from the PB of a CML patient in blast crisis. These cells possess a high proliferative potential (the efficiency index P/D = 2–12) and a significant content of СD34 ^+^ cells [6]. A moderate level of expression of *р53, mdm2, * and * bcl2 * with maxima on days 0.5, 6, and 9 and minima on days 2–4 and 11 corresponds to a wide proliferation and differentiation peak with the maximum peak of blast cells on days 1–3. Meanwhile, the concentration of immature cells is considerably higher than that of myelocytes. The peak of immature cells increases by days 5–8; however, by this time it mostly consists of myelocytes. The level of *bcr* expression rises, while *bcr/abl * expression decreases( *Figs. 2A–C* ).

High levels of *bcr/abl * expression( *Figs. 2А,B* ) with two maxima correspond to the profile of the P/D indices, as well as to the accumulation of blasts and myelocytes under proliferation and differentiation ( *Fig. 2C).* They also represent the beginning of cycles 1 and 2 of proliferation and differentiation with gene expression in early myeloid precursor cells [6].

Thus, the peak of the P/D index on day 1 and the distribution of gene expression in the range of *gapdh ~ bcr/abl > bcr >> p53 ~ mdm2 > bcl2* are typical mostly of blast cells (myeloid precursor cells consisting of ~75% blasts and promyelocytes). It can be seen that the expression level of *р53, mdm2, * and * bcl2* is fivefold lower than that of *bcr/abl* and *gapdh* . It is possible that either overexpression of *bcr/abl * and * gapdh* results in the inhibition of *р53, mdm2* ,and * bcl2;* or a decrease in the expression level of *р53 * and *mdm2 * causes uncontrolled division of Ph ^+^ cells.

The peak of the proliferation and differentiation of immature proliferating cells on day 7 includes mostly myelocytes; gene expression in the range *bcr >> gapdh >> p53 > bcl2 ~ mdm2 > bcr/abl * on days 4–6 is also determined by myelocytes. Gene expression in myelocytes and neutrophils subsequently decreases, which is in agreement with the low expression of a number of proteins and growth factors in neutrophils [51, 57, 64, 65, 68, 69].

On the other hand, it is known that protein BCR _(64-413)_ , overexpressed in Ph ^+^ cells in CML mice, is phosphorylated by the bcr/abl protein at the tyrosine residue, thus reducing the kinase activity of the bcr/abl oncoprotein by 80% [37–40]. Overexpression of *bcr* ( *Fig. 2* ) results in significant (but not complete) *bcr/abl* inhibition. The maximum of the expression peak of *bcr* is observed two days earlier than the maximum of the expression peak of *bcr/abl * and corresponds to high P/D indices = 6–12 and rapid development of a CML blast crisis in the patient [2].

A low level of p53 expression was also observed in the other Ph ^+^ cells during the acceleration phases and CML blast crisis phases with a high proliferative potential and a P/D index = 3–23. Thus, the level of *p53* expression on day 3 is no higher than that of * gapdh* . In these cells, the expression levels of *bcr/abl* , *mdm2* and *bcl2 * are comparable to that of *gapdh* , whereas the *bcr* expression level is twice as high. Ph ^+^ cells with a high P/D index (obtained from another CML patient) are characterized by a similar gene expression profile. These cells of the CML blast crisis may contain a defective *p53 * gene, although mutations in this gene are atypical of CML.

Thus, the composition and level of gene expression are different for type 1 Ph ^+^ cells with prolonged proliferation, the concentration of immature cells being higher than that of mature cells, and P/D index = 2–20. The cells with P/D index~ 5–20 are typically characterized by an increased content of blast cells (from CD34 ^+^ to promyelocytes) with overexpression of *bcr > gapdh > bcr/abl * and reduced expression of *р53, bcl2* and *mdm, р21< gapdh* . Activation of *bcr/abl * in myeloid precursor cells is accompanied by a low level of *p53, p21, * and * mdm2 * expression *. * The absence of a control performed by the genes regulating proliferation and the cell cycle presumably provides propitious conditions for the active proliferation of *bcr/abl ^+^* cells. These Ph ^+^ cells may also contain the mutant gene *р53* .

Type 1 Ph ^+^ cells with a low proliferative potential, P/D ~ 1.2–4, and content of immature cells higher than that of mature cells are characterized by a moderate *bcr/abl* expression with simultaneous overexpression of *p21, mdm2, p53, bcl2, * and * bax, * as well asproliferation and differentiation preferable for this Ph ^+^ clone. These genes participate in the regulation of the cell cycle; a wide peak on days 2–5 with a maximum on day 3 representing cell distribution over the S and G2/M phases of the cell cycle. This period is characterized by expression of the *p21, p53,* and * mdm2 * genes and interaction between p53 and mdm2, which mutually regulate each other’s expression.

Efficient proliferation with accumulation of immature cells and overexpression of *p21, p53 * and * mdm* 2 takes place in type 1 Ph ^+^ cells. Mature cells (neutrophils) formed during the period from day 3 to day 7 quickly enter apoptosis. The concentration of mature cells diminishes by almost an order of magnitude, which is an additional reason for the decrease in gene expression in the range *p21> mdm2> p53* . The aforementioned data is evidence of the fact that gene expression of *p21 > mdm2> p53* in the first zone of proliferation and differentiation (days 1–4) of type 1 Ph ^+ ^ cells ( *Figs.1 * and * 2* ) is 4–4.5 times higher than *gapdh* expression. On days 4–10, when the cell content in the S and G2/M phases is diminised significantly, the expression levels of these genes decrease by 3, 2.5, and 1.5 times as compared to those of *gapdh* , respectively. On days 8–9, the expression levels of these genes on the kinetic plot have a close minimum.

**Gene expression upon proliferation and differentiation of type 2 Ph ^+^ cells**

Significant accumulation of neutrophils (in particular, segments, which block apoptosis to a significant extent and inhibit the proliferation of Ph ^+^ cells) is typical of type 2 Ph ^+^ cells under the maturation stage. Proliferation and differentiation last for a long time and are characterized by low efficiency (P/D ^2^ ≤ 1), a higher maturation rate compared to the proliferation rate, and higher concentration of mature cells (neutrophils) compared to immature ones [1–4].

Type 2 Ph ^+ ^ cells * (Figs.3A–D) * were characterized by active expression of the *mdm2 > p53 * gene, a significantly weaker level of expression of *actin ~ gapdh> р21> bcr* *> с-myc ~ bcr/abl > bax > bcl2 * (a wide peak with a maximum on day 2), its duration and position of the maxima corresponding to increased (30–40%) cell accumulation in the S and G2/M phases for 3–4 days at a low apoptosis level (2–5%, *Fig. 3D* ). The expression levels of the *р21 > с-myc ~ bcr/abl > bax> bcl2* genes are lower than that of *gapdh* . By the time myelocyte production attains its maximum (days 4–5), expression of the *mdm2 > p53 * genes reaches its minimum (day 4). Meanwhile, neutrophil concentration was twice as high as myelocyte one during the entire observation time (5 days); according to [1, 3], this noticeably slows down the accumulation of immature cells and inhibits proliferation during days 1–5. Despite a higher level of neutrophil accumulation compared to the accumulation of immature cells with an identical time corresponding to their maxima and high cell content in the G2/M and S phases (~40%), the expression level of *mdm2 > p53 > gapdh * remains significant.

Under these conditions, the expression levels of *gapdh, actin, р21, bcr, с-myc, a* nd * bax* change negligibly; the level of *bcl2 * expression being no higher than half that of the *gapdh* level; this indicator being even lower for the other genes. Thus, despite the fact that the content of neutrophils and myelocytes is high, they have little impact on the expression of these genes. The expression levels of the *р21* , *bcr, с-myc, bcl2,* and * bax* genes in type 2 cells are 2 to 5-fold lower than those in type 1 cells. This allows to attribute overexpression of the * р53* and *mdm2* genes in type 2 Ph ^+ ^ cells to proliferating cells under the S and G2/M phases rather than to myelocytes and neutrophils under the maturation stage. The expression levels of *mdm2* and *р53 * under the S and G2/M phases in both cell types are similar and equal to 4.5 and 2–3 compared to those of *gapdh* .

The maximum levels of *bcr/abl * and * bcl2* expression (appreciably low) correspond to the maximum of the myelocyte peak. In the case of *bcr/abl, * the maximum corresponds to the highest myelocyte accumulation, an increase in the P/D index on day 4, and the maximum level of *bcr/abl > gapdh * expression on days 4–5. The *bcr/abl* expression decreases simultaneously with neutrophil accumulation and increases approximately two-fold, along with myelocyte production. The low levels of *bax * and * bcl2 * correspond to a low apoptosis percentage, in particular for *bcl2 > bax* , when apoptosis is blocked by *bcl2* . In other words, myelocytes and neutrophils are characterized by a low expression level of the *gapdh ~ actin > bcr, р21, bax, mdm2, p53* and * c-myc * genes, whereas the gene expression level of *bcr/abl * reaches its maximum in myelocytes ( *Figs. 3A–C* ).

**Gene expression upon proliferation and differentiation of type 3 Ph ^+^ cells**

Regulation of the proliferation and differentiation of type 3 Ph **+** cells depends on the order of alternation stages and the alternation scheme (1/2/1 or 2/1/2); i.e., what stage, proliferation (1) or maturation (2), is the first stage in the alternation. According to [1–4], proliferation and maturation are simultaneous processes; however, the rate of the preceding alternating stage is higher compared to the following one. The maximum proliferation rate corresponds to the minimum maturation rate, and vice versa. At the points where the accumulation plots of immature cells and neutrophils intersect, the rates of the stages are identical and their P/D is equal to 1. Thus, stage alternation determines the wave-like process of cell proliferation and differentiation. The alternation of stages according to schemes 1/2/1 or 2/1/2 differs not only by alternating rate decrease (either proliferation or maturation), but also in proliferation inhibition by high neutrophil concentrations under condition [mature] >> [immature] [1–4].

The character of gene expression in Ph **+ ** cells, as well as their proliferation and differentiation, depends on the order of the alternating stages and on the initial stage.

**Gene expression upon alteration of proliferation and maturation according to scheme 1/2/1**

*Figures. 4A–D* show that active gene expression coincides with the maxima of cell distribution during the G2/M + S phases and the maxima of the P/D indices (the maximum on days 2–3, *Figs. 4C,D* ). At the first stage (days 0–3), the proliferation and maturation rates differ negligibly (in terms of accumulation of immature cells and neutrophils) without a pronounced maximum ( *Fig. 4C* ). Approximately on day 3 (after intersecting the accumulation curves of immature and mature cells), the proliferation stage (Р/D = 1.4–1.1 and with a concentration of immature cells higher than that of mature cells) proceeds to the maturation stage (days 3–6) with maximum accumulation of neutrophils and their components (metamyelocytes, segments and bands) and decreasing efficiency index (P/D ^2^ < 1). Meanwhile, stage 2 is characterized by a significant (4-fold) increase in neutrophil concentration, attaining its maximum on day 5. The concentration of immature cells and myelocytes increases by only ~20%, also attaining its maximum on day 5. The amount of mature cells is three times higher than that of immature ones (low apoptosis level – 3–7%, *Figs. 4C,D* ). It can be seen that cell accumulation during the S phase on day 5 is accompanied by an insignificant increase in their apoptosis, which results in no increase in cell content in the G2/M phase ( *Fig. 4D* ). It is also clear that a 4-fold increase in the neutrophil content noticeably inhibits proliferation under the maturation stage.

It can be seen in *Figs. 4A–D* that the proliferation stage (1) on days 2–3 corresponds to the expression maxima of *mdm2 > p53 > bax > p21* , expression minima of *bcl2 > c-myc >> bcr/abl* , the first maximum of the S phase, and the maxima of G2/M, S+G2/M, and the P/D indices. The first maximum of *bcr/abl* expression, minima of *р53, mdm2, * and * bax* expression (maxima of mature >> immature > metamyelocytes > segments >> blasts, and rather low apoptosis maximum) corresponds to the expression of minima of *p53* > *p21* >> *bax* , *mdm2 * on days 4–5; cell minima in the G2/M, G2+S phases; P/D index on days 5–6; as well as the expression maxima of *bcr/abl * and * bcl2* . Meanwhile, the maxima of *c-myc* and G2+S and the second minimum of P/D on days 5–6, as well as the expression minima of *p53 ~ p21>> bax* , and G2/M minimum on days 4–5, correspond to peak 2 of the nonproductive S phase (not leading to the G2/M phase) on days 4–6.

Gene expression levels ( *Figs. 4A,B* ) at the first stage (days 2–3) decrease in the range *mdm2 >> p53 > bax ~ gapdh ~ p21 ~ bcl2 > bcr/abl* , whereas expression of the genes *bcl2, c-myc > bcr/abl * attains its minimum. Stage 2 (day 5) is characterized by expression maxima of *bcr/abl ~ bcl2 > gapdh* and an increase in the level of *actin, p53 ~ p21, * and * c-myc * at the minimum *bax* level. Overexpression of *mdm2 >> p53 >> bax * > *gapdh * (its maximum being observed on day 2) corresponds to the maximum cell content in the S and G2/M phases. After the end of proliferation and proceeding to the maturation stage, the expression of *р53 * and *mdm2 * decreases abruptly, whereas the *bcr/abl * and *bcl2 * expression increases. At the maturation stage, the maximum expression level (days 4–6) of the genes disposes into the following range: *bcr/abl ~ bcl2 > gapdh ~ actin ~ p21 ~ c-myc* **. ** The maximum levels of *bcr/abl * and *bcl2* expression are observed under insignificant accumulation of immature cells and myelocytes on day 5. Apoptosis is blocked (2–4% and no higher than 7% on days 5–6) upon expression of *bcl2* **>>** *bax* . This emphasizes the role of *bcl2* overexpression compared to low *bax* expression in such a significant suppression of apoptosis ( *Figs. 4A,D* ). In the case of *bcl2 > bax * or asynchronous maxima and minima of their expression, apoptosis inhibition was also observed in types 1 and 2 Ph ^+^ cells. An increase in the expression levels of a number of genes by days 5–6 can be regarded as a precursor of the proliferation stage, which follows the maturation stage.

During the proliferation stage, when the content of proliferating cells is just about higher than the neutrophil content, overexpression in the range *mdm2 >> р53> bax > gapdh * corresponds to the maximum of proliferating cells in the S and G2/M phases; small maxima of *bcr/abl* **~** *bcl2* **>** *gapdh * expression emerge during the maturation stage. The expression levels of the remaining genes are lower than that of *gapdh* during both the proliferation and maturation stages. The expression level of the *mdm2 * and * р53 * genes increases abruptly under the proliferation stage and rapidly decreases under the maturation stage in accordance with cell percentage in the G2/M phase. This means that *mdm2* expression is significant in proliferating cells and low or completely absent in neutrophils. Active *mdm2* expression can presumably act as a marker of the proliferation stage and cell activation of the cell cycle G2/M phase. The same character of change of the expression maxima of *mdm2, р53, * and * р21* coinciding with the cell maxima in the G2/M phase ( *Figs. 5* ) has also been observed under the maturation stage with the alternating scheme 2/1.

The *bcr/abl * expression is characterized by two maxima ( *Figs. 4А,B* ): the maximum of *bcr/abl ^1^ < gapdh * under the proliferation stage with the number of immature cells being insignificantly higher than that of mature cells. However, the maturation stage (upon high concentration of mature cells, their content being significantly higher than that of immature cells) is characterized by a maximum expression level in the range *bcr/abl ^2 ^ > gapdh * and *bcr/abl ^1^* **<** *bcr/abl ^2^* ( *Figs. 4A* –C). Let us note that *bcr/abl* expression also increases with decreasing GEL of *р53, mdm2* , and *p21 * upon proliferation and maturation of Ph ^+^ cells according to the alternating scheme 1/2. In types 2 and 3 Ph ^+^ cells, the expression level is *bcr/abl ^1^* *< bcr/abl ^2 ^* ( *Figs. 3* and *4* ). However, the range of *bcr/abl ^1^ < bcr/abl ^2^ , * and *bcr/abl ^1^ > bcr/abl ^2^* can also occur in the type 1 Ph ^+^ cells.

Thus, gene expression correlates with regulation of the proliferation and differentiation of type 3 Ph ^+^ cells with alternation of the proliferation and maturation stages according to scheme 1/2. In this case, the increased expression level of *р53, mdm2, * and *р21 * coincides with the maximum of the S+G2/M phases and corresponds to a low level of *bcr/abl * expression.

**Gene expression in Ph ^+^ cells with stage alternation according to scheme 2/1/2**

When the proliferation and maturation stages were alternated according to schemes 2/1–2/1/2/1, sequential changes in the concentration of type 3 Ph ^+^ cells were observed in the following range: [mature] > [immature] → [immature] > [mature] → [mature] > [immature] ( *Figs. 5–9* ).

It is clear from *Figs.*   *5* and *6* that gene expression levels upon maturation and proliferation correspond to a low content of proliferating cells in the phases of the cell cycle (10–20%), whereas apoptosis induction is significant (40–80%). Meanwhile, a high content of neutrophils that are incapable of dividing results in a decrease in the proliferative cell pool in the S+G2/M phases, which is particularly noticeable in *Fig. 6* . This pool does not increase upon proliferation on days 2–6. The cell maximum in these phases does not presumably coincide with a significant accumulation of immature cells during the proliferation stage. However, neutrophils maturating without division naturally decreases the accumulation of proliferating cells in the S and G2/M phases. Meanwhile, gene expression in neutrophils is significantly diminished, and gene expression with increased activity occurs only in the proliferating cell pool of the S + G2/M phases. Thus, neutrophil (mature cells) accumulation resulted in decreasing gene expression.

The expression levels of the genes under investigation are considerably lower here compared to the aforementioned examples, including the expression level compared to *gapdh* .

Upon proliferation and differentiation of Ph ^+^ cells starting with the maturation stage, along with a significant level of neutrophil accumulation and proliferation inhibition on days 0–3 ( *Figs. 5* and *6* ), the maximum of neutrophil accumulation corresponds to the minima of the efficiency index P/D and accumulation of immature cells and myelocytes. When proceeding to the proliferation stage on days 3–5, the minima of accumulation of neutrophils (mature cells), a decrease in P/D, and minima of neutrophil accumulation become clear. The concentrations of mature and immature cells in their maxima differ by 4–5 times, which allows one to attribute gene expression to the neutrophils or myelocytes that are incapable of dividing.

Under the maturation stage ( *Figs. 5A* –D), the maximum expression of *р21, mdm2, p53 > bcl2, >bax * on day 2 characterizes proliferating cells in the S and G2/M phases (20%) rather than neutrophils, since a 5-fold increase in myelocyte accumulation during the proliferation stage on day 5 results in a decrease in the expression level of these genes to the minimal values.

The maximum expression level of *bcr/abl* , *actin, gapdh* *c-myc * observed on day 5 characterizes myelocytes (P/D ^2^ = 2.5). Two peaks of *bcr/abl* expression (compared to *gapdh, Figs. 5A,B* ) upon myelocyte proliferation are twice as high as those upon neutrophil maturation (days 5 and 0.5). The minima of gene expression *gapdh > actin > bcr/abl* can also be seen during the maturation stage (on day 2). This means that expression of the genes regulating the cell cycle in proliferating immature cells is also activated during the maturation stage in accordance with the cell maximum in the S and G2/M phases; however, the expression level is 2- to 3-fold lower than that in types 1 and 2 Ph ^+^ cells.

The expression levels of the genes *р21 ~ mdm2 ~ p53 > gapdh* in *Fig. 5* are higher than those in *Fig. 6* . *Fig. 6* demonstrates that at the maturation stage, the cell content in the S + G2/M phases is twice as low, and that neutrophil content is five times higher than that of immature cells, whereas the content of segments is considerably higher. In other words, increasing neutrophil content results in low content of cells accumulated in the S and G2/M phases and a decrease in the relative levels of expression of the *р21, mdm2, p53* , and *gapdh* genes ( *Figs. 5* and *6* ).

Under the proliferation stage with a maximum peak of myelocytes (on day 5) only for the *bcr/abl * and * actin* genes, the expression levels are higher than those of *gapdh* , whereas the expression levels for *p53 > c-myc > bax > mdm2 > p21 * are lower. Two maxima of *gapdh * expression correlate with the apoptosis maxima ( *Figs. 5A,B,D* ) **.** *Figures 6A,B,D* demonstrate that only *р53* and *bcl2 * are characterized by a more active expression on days 2–4 compared to *gapdh* . The maximum level of expression of genes *р53 > gapdh >> mdm2 > p21 * on days 2–4 also corresponds to the maximum of the wide apoptosis peak (on days 2–5). This differs from the moderate * gapdh* expression in previously discussed examples of proliferation and differentiation without stage alternation and can be presumably attributed to the participation of *gapdh* in apoptosis induction with the maxima on days 1 and 5. Let us also note that expression of *p53, c-myc * and *bcl2* , which is minimal at the proliferation stage on days 3–6, is equal to 0.5–0.7 of the maximum level of *gapdh * expression( *Fig. 6* ). It is clear from Fig. 6 that the expression maxima of the *р53 > mdm2 > p21 * genes on days 2–4 also correspond to the maximum of a wide apoptosis peak (on days 2–5).

It is known that the expression of *p21, p53, gapdh* , and *c-myc * can be responsible for apoptosis induction [13–16, 20, 21, 28, 55, 56]. At the proliferation stage on days 3–6 in the absence of *bax * and * bcl2 * expression, apoptosis is apparently induced by the *gapdh, p21, * and *p53* genes * (Figs. 5* and *6* ). Let us note that the level of *bcr/abl* expression on day 0.5–1 corresponds to a maximum short-term accumulation of myelocytes and immature myelocyte precursor cells. Gene expression, attaining its maximum by day 0.5, changes in the range *р53 > gapdh > actin > bcr/abl* **. ** The level of *bcr/abl * expression on days 0–1 is twice as low as that on day 5, which is also caused by proliferation inhibition in Ph ^+^ cells at an increased neutrophil concentration ( *Figs. 5 and 6* ).

Thus, the relative changes in the gene expression levels in Ph ^+^ cells correspond to stage alternation according to scheme 2/1 (from maturation to proliferation). Gene expression is in agreement with the inhibition of proliferation in immature cells by neutrophils maturating without dividing. Gene expression under the maturation stage with a maximum content of neutrophils (in the form of segments under a small content of proliferating cells in the S and G2/M phases) is several-fold lower than that in types 1–3 Ph ^+^ cells with a maximum proliferative cell pool. In these cases, there is an unambiguous increase in gene expression in actively proliferating cells during the S and G2/M phases, whereas neutrophils as nondividing cells are absent in these phases.

A low expression level of the genes studied in neutrophils can be seen under the maturation stages of types 2 and 3 Ph ^+^ cells ( *Figs. 3–6* ), which agrees with the diminished production of a number of proteins and growth factors in neutrophils [51, 57, 64, 65, 68, 69].

It can be seen ( *Fig. 6* ) that the expression levels of all genes in type 3 cells with the alternating 2/1 stages are diminished under maturation stage upon increased neutrophil content. The expression levels of the *p21 ~ mdm2 ~ p53 > gapdh* *> c-myc * genes under the maturation stage are 3–5-fold lower compared to the proliferation stage ( *Figs. 5 and 6* ). The character of *р21 * and * mdm2* expression is altered. The peaks of expression of these genes, which attains its maximum on days 1 or 2, become narrower, followed by a decrease to the minimum value, along with termination of the S and G2/M phases of the cell cycle.

It should be noted that *bcr/abl * expression considerably increases under proliferation stage with myelocyte accumulation. The *bcr/abl* expression during the neutrophil maturation stage is twice as low as during myelocyte accumulation. The *bcr/abl * expression during the proliferation stage depends on the type and concentration of proliferating myeloid precursor cells (blasts), in which *bcr/abl * expression is presumably suppressed by active expression of *р53, mdm2, * and *p21* . In addition to proliferation inhibition in type 3 Ph ^+^ cells with alternating scheme 2/1, inhibition of *bcr/abl* expression to its minimum (1.5–3 times lower than that of *gapdh* ) occurs under the maturation stage ( *Figs. 5–6). * Meanwhile, *gapdh* and *actin* are the only genes that are noticeably expressed in neutrophils in the maturation maximum (days 1–2, segments being the major components). The minimum levels of *c-myc, bcr/abl, p53 > p21 > bcl2 > bax* are 2–10 times lower compared to those of *gapdh* ( *Fig. 6A,B* ), which is in agreement with the low cell content in the S and G2/M phases (< 12%).

Upon prolonged alternation of the 2/1/2/1 stages for Ph ^+^ cells with a very low cell content in the S and G2/M phases (2–5%) and active apoptosis, gene expression also correlates with the alternation of the maturation and proliferation stages. Expression of *bcr/abl > gapdh* ≥ *c-myc * genes is increased, whereas the expression level of the *mdm2, p53, and bcl2 * genes remains low in both the maturation and proliferation stages ( *Figs. 7A,B* ). The *bcr/abl* expression is characterized by two peaks that are larger than those for the *gapdh* and * bcr/abl* ^1 ^ > * bcr/abl* ^2 ^ genes under the maturation and proliferation stages, respectively (Fig. 7). The maturation stage with a high level of neutrophil accumulation is accompanied by the expression of *bcr/abl* > *gapdh* > *c-myc * > *p53* > *mdm2* , which approaches a minimum by day 5. Under the proliferation stage (days 5–7), the expression levels increase again to their maximum value (on days 7–8), to decrease subsequently with a clear order. Thus, the maxima and minima of Ph ^+^ cell accumulation during the maturation and proliferation stages alternate in the same manner as the maxima and minima of gene expression in the range *c-myc* , *bcr/abl, gapdh* , *р53. * They correspond to high levels of *bcr/abl * and * c-myc * expression and very low levels of *bcl2* and *mdm2 * expression *. * Rather quick neutrophil accumulation induces suppression of immature cells proliferation, expression of their genes, and a decrease in cell content in the S and G2 phases to 3–5% ( *Figs. 7C,D* ). The sequence of these events affects the gene expression level in the range *c-myc* ~ *gapdh * ~ *bcr/abl > р53 > mdm2 * on days 1–9 ( *Figs. 7A–D* ).

Two maxima of gene expression can be seen in another example of BM-derived Ph ^+^ cells ( *Fig. 8* ) with alternating 2/1/2/1 stages and an increased cell content in the S and G2 phases (~ 30%, with two maxima on days 2 and 6): the first maximum corresponds to the maturation stage ( *р21* > *bax* ~ *c-myc* ~ *actin* >> *bcr/abl * and *gapdh ~ bcl2 ~ p53 * > *mdm2* ), and the second maximum corresponds to the proliferation stage (c- *myc ~* *р21* > *bax* >> *bcr/abl* > *actin* and *gapdh * > *mdm2 > p53 > bcl2* ) *. * The second maximum of the expression levels of *р21* > *bax* ~ *c-myc * is an order of magnitude higher than the first one. In Ph ^+ ^ cells derived from PB cells ( *Fig. 9* ) isolated from the same CML patient, the expression of *р21* > *bax* ~ *c-myc* genes was significantly lower than the expression levels in BM-derived cells ( *Fig. 8* ) and remained high under the proliferation stage after thrice-repeated accumulation of PB neutrophils during the maturation stage. In other words, a significant level of neutrophil accumulation suppresses gene expression during the maturation stages even if the cell content in the S + G2/M phases is increased. 

The results shown in *Figs. 7–9* are notable for the fact that *Fig. 7* demonstrates the effect of a long-term excess of neutrophils over immature cells on gene expression and complete suppression of the proliferating cell pool in the S + G2/M phases under a low level of apoptosis. *Figures 8–9* show the suppression of gene expression by neutrophils at the maturation stage, almost coinciding with the cell maxima in the S + G2/M phases (30%). However, proceeding to proliferation with a significant accumulation of immature proliferating cells under conditions of 50–80% apoptosis induction (which previously was 10–20%) results in the formation of a second maximum corresponding to the accumulation of the proliferating pool in the S + G2/M phases. Expression of the *р21* > *bax* ~ *c-myc* > *bcr/abl > mdm2* genes increases by an order of magnitude at the minima of *p53 > bcl2 * expression. In other words, neutrophils are capable of suppressing and delaying the formation of the proliferating cell pool in phases of the cell cycle and/or suppressing the expression of proper genes. These terms can also be used to interpret the results shown in *Figs. 4–6* .

Thus, gene expression in neutrophils and myelocytes under proliferation and differentiation with stages alternating according to the scheme 2/1 – 2/1/2/1 is in agreement with the types of cell regulation by stage alternation, apoptosis, and distribution of CML Ph ^+ ^ cells in the cell cycle phases. This provides additional support to the argument that neutrophils block apoptosis and inhibit Ph ^+ ^ cell proliferation. The gene expression levels under the maturation stages are determined by the maximum level of cell accumulation in the S and G2/M phases of the cell cycle and by inhibition of proliferation by neutrophils. The coincidence of the maxima of cell accumulation in the S + G2/M phases and during the proliferation stage attests to their contribution to the 1.5–7-fold rise in the expression levels of *р21,* *mdm2* , *p53* , *bax, c* - *myc* .

The expression levels of the other genes in neutrophils under the maturation stage are 2- to 10-fold lower than that of *gapdh * gene. This expression level is comparable to those in type 2 cells and is 5- to 10-fold lower than the expression levels in type 1 immature cells.

## DISCUSSION

The kinetic curves of the expression of 10 genes that regulate the proliferation and differentiation, the cell cycle, and apoptosis were determined in hematopoietic cells containing the Ph chromosome and the *bcr/abl* oncogen, which were derived from CML patients. The expression of the main cell cycle regulators ( *р53, mdm2, p21, c-myc, bcr/abl, bax, bcl2, * and *gapdh* ) in differentiating proliferating myeloid Ph ^+^ cells and neutrophils maturating without dividing correlates with the regulation of proliferation and differentiation processes, with apoptosis induction, and distribution in the phases of the cell cycle * ex vivo* . It has been demonstrated by comparing the kinetics of gene expression and regularities of the regulation of the proliferation and differentiation of Ph ^+^ cells *ex vivo* with the functions of these genes that the genes participate in the regulation of the proliferation and differentiation of three main types of Ph ^+ ^ cells, as well as in the alternation of the proliferation (1) and maturation (2) stages.

The gene expression levels can be regarded as estimates that are only demonstrating the general trend, since the RT-PCR data were compared with the expression levels of the *gapdh* and *actin* genes, which can be changed themselves (measured in the same probes) upon cultivation instead of using internal reference standards for each individual gene.

It has been revealed that gene expression changes synchronously with proliferation and differentiation regulation, cell cycle phases, and apoptosis. This fact demonstrates that the genes under consideration participate in the regulation of the proliferation and differentiation of proliferating myeloid Ph ^+ ^ cells and maturating neutrophils. The results obtained are in agreement with the available data pertaining to the regularities of these genes’ expression in other cells. The data also correspond to the regularities of proliferation and differentiation, cell cycle, and apoptosis in other systems. This attests to the fact that these methods and the kinetic plots obtained by RT-PCR can be used to study gene expression. A low level of gene expression in neutrophils is in agreement with low production of the p21 protein, a number of specific proteins, and a number of factors in haematopoietic neutrophils [51, 57, 64, 65, 68, 69].

The kinetic approach to the study of gene expression using RT-PCR by comparison with the kinetics of cell proliferation and differentiation in a polynomial approximation appears to be a rather informative approach to investigating the regulation of proliferation and differentiation, the cell cycle, and the apoptosis of haematopoietic cells proliferating with differentiation and maturing without dividing. The results obtained allow one to ask new questions that are important for gaining further insight into the gene expression and CML mechanisms. One such question is whether the *р53, mdm2, p21, * and * c-myc* genes participate in the inhibition of *bcr/abl* expression. The second question is whether *bcr/abl * expression is genotoxic or cellular stress for hematopoietic cells and what is the response of the *р53* , *mdm2, p21, * and *c-myc* genes.

The results obtained in this study indicate that a diminished expression of the *p53, mdm2, * and * р21 * genes,whichcreates conditions for the uncontrolled expression of *bcr/abl* , promotes an increase in the rate of proliferation and aggressiveness of proliferating Ph ^+^ cells with a high level of *bcr/abl * expression. On the contrary, overexpression of the *р53* , *p21, mdm2,* and * c-myc* genes (the major cell cycle regulators) presumes suppression of *bcr/abl * expression in Ph ^+^ cells and formation of *bcr/abl ^+^* cells.

## CONCLUSIONS

1. Expression of the *p53, mdm2 * and * p21, c-myc,* *bcr/abl, bcr, bcl2, bax, gapdh, actin* genes contributes to the total program of *ex vivo* regulation of the proliferation and differentiation of CML Ph ^+^ cells.

The expression of these genes is in agreement with the proliferation and differentiation of Ph ^+^ cells of three types and their regulation via alternation of the proliferation (1) and maturation (2) stages according to the schemes 1/2/1 and 2/1/2 and with proliferation and differentiation at either the proliferation (type 1) or maturation (type 2) stage.

2. The *p53, p21, mdm2 >> gapdh * genes are overexpressed in the actively proliferating myeloid precursor cells accumulating in the S and G2/M phases of the cell cycle. Overexpression of these genes is observed in type 1 cells and when the cell maximum during the S and G2/M phases coincides with the proliferation stage in types 2 and 3 Ph ^+ ^ cells. Gene expression is significantly diminished upon maturation and repeated alternation of the proliferation and maturation stages, where neutrophils and myelocytes are accumulated. Alternating according to scheme 2/1/2 results in a decrease in cell content in the S and G2/M phases of the cell cycle.

3. The expression level in neutrophils under the maturation stage decreases in the range *gapdh > actin > c-myc, bcr/abl, p21 > p53 > bcl2 > bax* ; the expression level of these genes in myelocytes is also lower than the expression level of *gapdh* .

4. Expression of the *bcr/abl * gene in types 2 and 3 Ph ^+^ cells has two peaks, decreasing under the maturation stage as apoptosis is blocked and neutrophils accumulate and increasing 2- to 3-fold under the proliferation stage with myelocyte accumulation. Overexpression of the *p53, mdm2, p21,* and * c-myc* genes and cell maximum in the S and G2/M phases of the cell cycle correspond to a minimum level of *bcr/abl* expression.

5. The maturation stage involves apoptosis inhibition, neutrophil accumulation, and a decrease in the expression level of the *p53, mdm2 * and *p21, c-myc, * and *bcr/abl * genes *.* Apoptosis in Ph ^+ ^ cells is induced by gene expression of *bax > bcl2, р53, p21, c-myc * and * gapdh* .

6. Overexpression of the genes *bcr > gapdh > bcr/abl * and diminished expression of *р53, bcl2, mdm, р21 < gapdh* are observed in type 1 Ph ^+^ cells derived during the blast crisis and the CML acceleration phase with the efficiency indices P/D ~ 5–20 and a high CD34 ^+ ^ cell content. Overexpression of *bcr/abl* in myeloid precursors is accompanied by low expression of the *p53, p21, mdm2 * genes. It was assumed that the decrease or absence of control over the genes encoding the regulators of proliferation, differentiation, and the cell cycle promotes *bcr/abl * overexpression and active production of *bcr/abl* **+** cells.

## Abbreviations

GEL: gene expression level
CML: chronic myeloid leukemia
Ph^+^cells: hematopoietic cells containing Ph chromosome
PB: peripheral blood
BM: bone marrow
FBS: fetal bovine serum
RT-PCR: reverse transcription polymerase chain reaction
CPD: cell proliferation and differentiation
